# Pleiotropic effects of a single gene on skeletal development and sensory system patterning in sticklebacks

**DOI:** 10.1186/2041-9139-5-5

**Published:** 2014-02-05

**Authors:** Margaret G Mills, Anna K Greenwood, Catherine L Peichel

**Affiliations:** 1 Divisions of Human Biology and Basic Sciences, Fred Hutchinson Cancer Research Center, 1100 Fairview Avenue North, Seattle WA, 98109, USA; 2 Graduate Program in Molecular and Cellular Biology, University of Washington, 1959 NE Pacific Street, Health Sciences Building T-466, Seattle 98195, WA, USA

**Keywords:** Pleiotropy, Genetics of adaptation, Lateral line, Neuromast, Dermal skeleton

## Abstract

**Background:**

Adaptation to a new environment can be facilitated by co-inheritance of a suite of phenotypes that are all advantageous in the new habitat. Although experimental evidence demonstrates that multiple phenotypes often map to the same genomic regions, it is challenging to determine whether phenotypes are associated due to pleiotropic effects of a single gene or to multiple tightly linked genes. In the threespine stickleback fish (*Gasterosteus aculeatus*), multiple phenotypes are associated with a genomic region around the gene *Ectodysplasin* (*Eda*), but only the presence of bony lateral plates has been conclusively shown to be caused by *Eda*.

**Results:**

Here, we ask whether pleiotropy or linkage is responsible for the association between lateral plates and the distribution of the neuromasts of the lateral line. We first identify a strong correlation between plate appearance and changes in the spatial distribution of neuromasts through development. We then use an *Eda* transgene to induce the formation of ectopic plates in low plated fish, which also results in alterations to neuromast distribution. Our results also show that other loci may modify the effects of *Eda* on plate formation and neuromast distribution.

**Conclusions:**

Together, these results demonstrate that *Eda* has pleiotropic effects on at least two phenotypes, highlighting the role of pleiotropy in the genetic basis of adaptation.

## Background

Adaptation to a new environment often requires changes in numerous phenotypic traits [1-3]. Mechanisms that lead to the co-inheritance of suites of phenotypes might therefore facilitate adaptation [4-6]. Indeed, in many cases of adaptation to divergent habitats, experimental evidence demonstrates that multiple phenotypic traits map to the same genomic regions [7-15]. However, whether phenotypes that co-vary are caused by allelic variation in the same gene (that is, pleiotropy) or in tightly linked genes is only known in a handful of cases because identification and experimental manipulation of the gene(s) are required [16-23]. The extensive phenotypic diversification of threespine stickleback fish (*Gasterosteus aculeatus*) in freshwater habitats provides a tractable system to address this question [24]. Marine and freshwater sticklebacks from independently derived populations have diverged in a suite of physiological, morphological and behavioral traits [25,26]. Many phenotypic traits and measures of fitness that differ between stickleback populations have been linked to a single genomic region surrounding the *Ectodysplasin* gene (*Eda*) on chromosome 4 [27-38]. However, only a single trait - bony lateral plates - has actually been demonstrated to be controlled by *Eda*[39].

**Figure 1:**
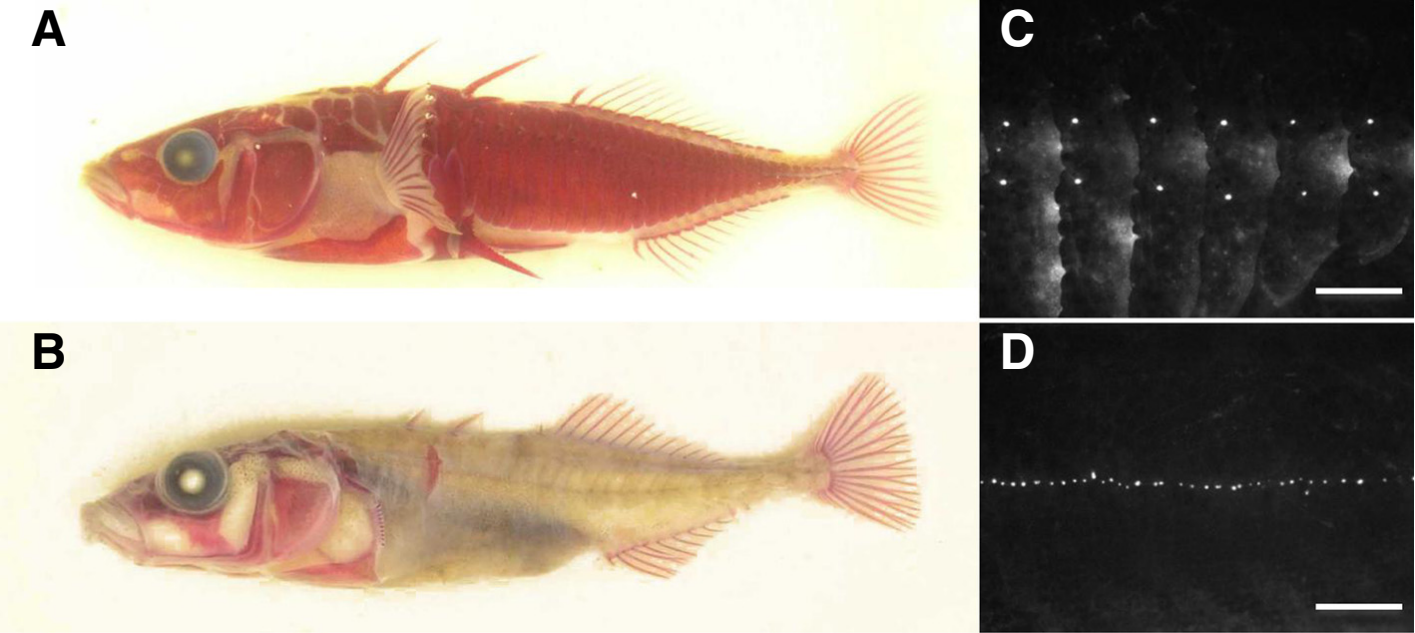
**The presence of bony lateral plates is associated with variation in neuromast distribution in stickleback populations. (A)** Alizarin red stained Japanese Pacific (JP) marine stickleback with a complete set of bony lateral plates. **(B)** Alizarin red stained Paxton Benthic (PB) freshwater stickleback with a single bony lateral plate. **(C)** Close-up of the Mp line of a DASPEI stained Japanese Pacific (JP) marine stickleback, highlighting the dorso-ventral distribution of neuromasts on the lateral plates. **(D)** Close-up of the Mp line of a DASPEI stained Paxton Benthic (PB) freshwater stickleback, highlighting the placement of the neuromasts in a continuous row along the midline and the presence of multiple neuromasts per body segment. Scale bars = 0.1 cm. DASPEI, 2-(4-(dimethylamino)styryl)-N-ethylpyridinium iodide; JP, Japanese Pacific Ocean marine; Mp, main trunk line, posterior; PB, Paxton benthic.

**Figure 2:**
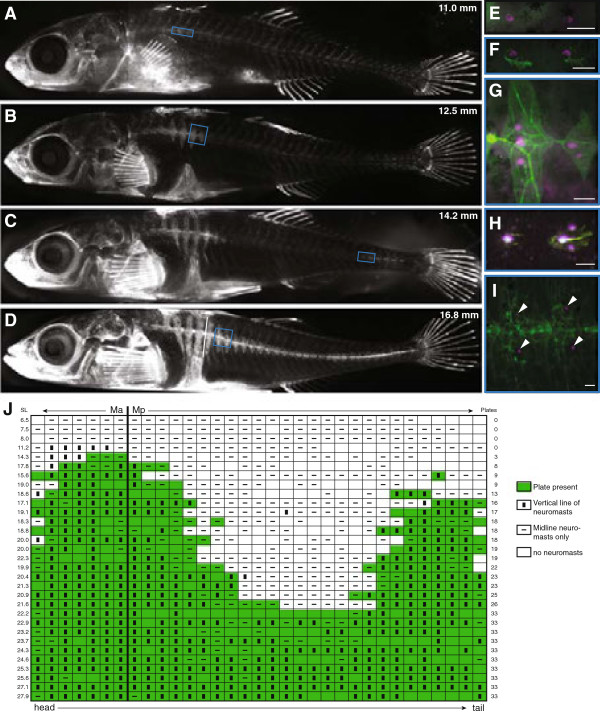
**Bony plates and neuromast distribution are correlated through development in completely plated fish. (A-D)** Bone development in a single Japanese Pacific (JP) marine fish, repeatedly stained with calcein during development. The standard length of the fish is indicated in mm. The near-vertical white dotted line in **(D)** indicates the location of the boundary between the Ma and Mp lines. **(E-I)** Detail of neuromast and plate development along the posterior flank of DASPEI- and calcein-stained JP fish (scale bar = 0.1 mm); **(F-I)** are from similar portions of the flank as the boxed regions in **(A-D)**, although the pictures are from different fish. The white arrowheads in **(I)** indicate the location of neuromasts. **(J)** Compilation of plate presence and neuromast distribution along the length of Manchester Clam Bay (MC) marine fish at a range of sizes, showing the correlation between plate development and neuromast elaboration. Each horizontal row is a single individual fish; fish are sorted in order of total number of plates. Standard length is indicated in mm. The vertical line between columns seven and eight indicates the location of the boundary between the Ma and Mp lines. Green shading indicates the presence of a plate, white indicates no plate, an unfilled box indicates no neuromasts present, a horizontal line indicates neuromasts along the midline, and a vertical box indicates dorso-ventral distribution of neuromasts. DASPEI, 2-(4-(dimethylamino)styryl)-N-ethylpyridinium iodide; JP, Japanese Pacific Ocean marine; Ma, main trunk line, anterior; MC, Manchester Clam Bay marine; Mp, main trunk line, posterior; PB, Paxton benthic.

**Figure 3:**
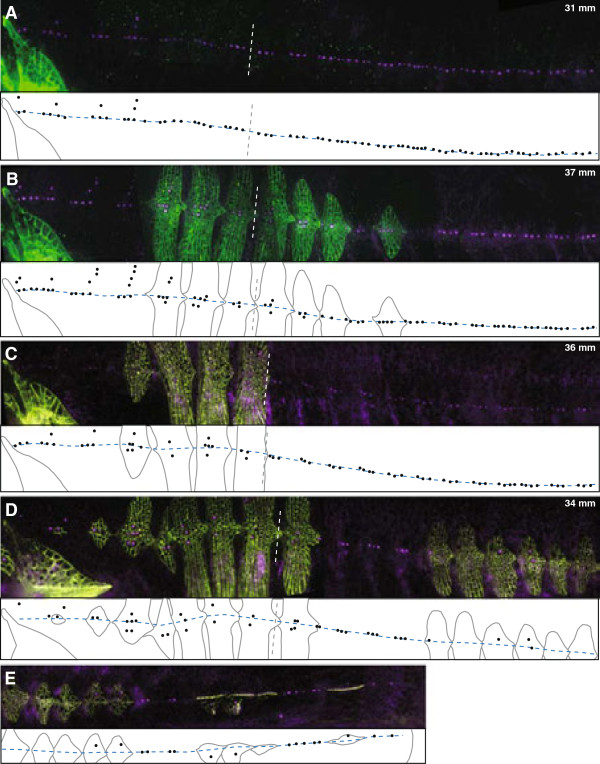
**Transgenic expression of *Eda *increases number of plates and alters neuromast distribution. (A-E)** Plates were visualized with calcein (green) and neuromasts were visualized with DASPEI (purple) on representative control and transgenic fish from PB and PB x JP low plated F3 backgrounds, the Ma/Mp boundary is shown (white dotted line); schematics below each image show plate edges (gray lines), neuromast placement (black dots), Ma/Mp boundary (vertical or near-vertical gray dotted line) and the midline (blue dashed line). For all fish, dorsal is up and rostral is to the left, with the shoulder girdle (cleithrum) appearing to the left of the first Ma segment. The standard length of each fish is indicated in mm. **(A)** PB control; **(B)** PB transgenic; **(C)** PB x JP low plated F3 control; **(D)** PB x JP low plated F3 transgenic; **(E)** Remainder of the flank from the individual shown in (**D**). Individual frames comprising **(B)** were tilted slightly with respect to each other to correct for bend in fish body during photographing. DASPEI, 2-(4-(dimethylamino)styryl)-N-ethylpyridinium iodide; JP, Japanese Pacific Ocean marine; Ma, main trunk line, anterior; Mp, main trunk line, posterior; PB, Paxton benthic; PB x JP F3, Paxton benthic x Japanese Pacific Ocean marine F3 hybrid.

Here, we ask whether pleiotropy or linkage is responsible for the correlation between lateral plate presence and another trait: the distribution of the neuromasts of the lateral line [38]. The lateral line sensory system in aquatic vertebrates detects water motion and has been shown to play a role in schooling, predator avoidance, prey capture, and other behaviors [40-45]. The lateral line system comprises clusters of sensory hair cells called neuromasts, which are arranged in distinct lines on the body and head. Threespine sticklebacks possess twelve lines of superficial neuromasts: nine on the head, two on the trunk, and one at the base of the caudal fin [46]. The number of neuromasts in each line varies in sticklebacks from different habitats in parallel with the environment [46], suggesting that the differences among populations might be adaptive. Lateral line variation across stickleback populations is particularly striking in the lines along the trunk, both in the number of neuromasts and in the distribution of those neuromasts. Specifically, variation in neuromast distribution is correlated with the presence of bony lateral plates (Figure 1) [46], a trait that varies consistently and predictably in marine versus freshwater environments [47-49]. Neuromasts on the trunk of sticklebacks can be separated into two anatomical regions [46]: those located in body segments anterior to the second dorsal spine are considered ‘Ma’ (main trunk line, anterior) and those located in body segments posterior to this boundary are called ‘Mp’ (main trunk line, posterior; Figure 2D). Marine sticklebacks, like those from the Pacific Ocean in Japan (‘JP’) or Manchester Clam Bay in Washington State, USA (‘MC’), have a complete set of bony plates along their entire flank, in body segments coincident with both Ma and Mp (Figure 1). They typically have a vertical line of neuromasts, with pairs of neuromasts (one dorsal and one ventral to the midline; hereafter called a ‘dorso-ventral’ distribution) on each of the plates coincident with Mp and clusters of multiple vertically arranged neuromasts on each of the plates coincident with Ma [38,46]. Most freshwater populations also have bony plates in the region of the Ma line, and these plates possess a dorso-ventral distribution of neuromasts. However, most freshwater sticklebacks are low plated; that is, they have no bony plates in the region of the Mp line, and each body segment in this region has a single neuromast that is located along the midline [46]. Several freshwater populations, including benthic sticklebacks from Paxton Lake in British Columbia, Canada (‘PB’), exhibit striking reduction in plate number, with very few plates in the region of the Ma line in addition to a complete lack of plates in the region of the Mp line. The neuromasts in both the Ma and Mp lines are found in a nearly continuous row along the midline (Figure 1), although there are a few neuromasts dorsal to the midline in the Ma line even in the absence of plates (Figure 3A). These fish also exhibit extra trunk neuromasts, with up to five neuromasts per body segment [38,46].

Our previous quantitative trait locus (QTL) mapping study of variation in the lateral line between JP and PB sticklebacks uncovered tight genetic linkage between neuromast distribution and lateral plate presence in the Mp line [38]. The presence of plates in the Mp region was strongly associated with a dorso-ventral distribution of neuromasts in F2 hybrids, and both the number of plates and neuromast distribution in the region of Mp (but not Ma) mapped to a region of chromosome 4 containing the gene *Eda*. This gene was previously shown to be responsible for the difference in plate number between complete and low plated sticklebacks [39]. We focus here on dissecting the developmental and genetic relationship between *Eda*, lateral plates, and neuromast distribution in the Mp line.

## Methods

### Fish care

All animal protocols were approved by the Fred Hutchinson Cancer Research Center Institutional Care and Use Committee (protocol #1575). All sticklebacks were lab reared from *in vitro* crosses of fish from previously studied populations [38,46]: marine sticklebacks from Manchester Clam Bay, Washington, USA (‘MC’) and from the Pacific Ocean, Akkeshi, Japan (‘JP’); and benthic sticklebacks from Paxton Lake, British Columbia, Canada (‘PB’). Fish were housed in 29-gallon glass aquaria with independent filtration and air stone aeration. Water in each tank contained 3.5 g/L Instant Ocean salts (Instant Ocean, Cincinnati, OH, USA) and 0.4 g/L NaHCO_3_. The fish were kept at summer conditions of 15.5°C on a 16 h light/8 h dark cycle. Eggs and fry were contained in net breeders suspended from the side of the tank until 2 weeks after hatching. Young fish were fed brine shrimp nauplii twice daily, and adults were fed brine shrimp nauplii and frozen Mysis shrimp.

### Imaging of plates and neuromasts

To visualize neuromasts, live fish were placed in 0.025% 2-(4-(dimethylamino)styryl)-N-ethylpyridinium iodide (DASPEI; Invitrogen/Molecular Probes, Carlsbad, CA, USA) in zebrafish embryo medium (1 mM MgSO_4_, 120 μM KH_2_PO_4_, 74 μM Na_2_HPO_4_, 1 mM CaCl_2_, 500 μM KCl, 15 μM NaCl, 500 μM NaHCO_3_) for 15 minutes, rinsed with clean embryo medium, then anesthetized with fresh 0.013% MS-222 (Tricaine methanesulfonate; Argent Chemical Laboratories, Redmond, WA, USA) in embryo medium, adjusted to pH 7.2 with NaOH (Fisher Scientific, Pittsburgh, PA, USA). To visualize calcified bone, live fish were placed in 0.2% calcein (Sigma-Aldrich, St. Louis, MO, USA) in embryo medium for 10 minutes, washed twice with clean embryo medium, then anesthetized as after DASPEI staining. For double staining with calcein and DASPEI, fish were stained first with calcein, returned to their tanks, and then stained with DASPEI one to two days later. Staining with both calcein and DASPEI was visualized using fluorescence microscopy (see below).

To describe the developmental processes by which the adult lateral line pattern is produced in completely plated populations, we followed clutches of marine (JP and MC) fish through development. We first used repeated calcein staining in JP fish to visualize calcified bone, which allowed us to characterize patterns of plate formation in individuals. Fish used for repeated imaging were kept in individual plastic cups starting at one week post hatching, in 100 ml of zebrafish embryo medium that was changed daily. For approximately two months they were stained once a week with calcein, lightly anesthetized, photographed under the microscope, and returned to their cups.Unfortunately, we could not perform repeated DASPEI staining to visualize neuromasts on the same individuals because this leads to a loss of reliable neuromast staining over time (MM, personal observation). Thus, we also stained an additional set of fish at a range of sizes after hatching; each individual fish was simultaneously stained with DASPEI and calcein, and then not stained again. Plate and neuromast phenotypes were scored as follows: within each body segment (generally myomeres), any calcein-stained bone (including the first sliver to appear around the central neuromast, Figure 2F) was scored as a plate; segments were scored as ‘dorso-ventral’ if they contained any neuromasts above or below the midline or ‘midline’ if they contained neuromasts located exclusively on the midline. The midline of neuromast placement was determined based on the midline ridge of plates (in plated segments) or in relation to the surrounding neuromasts (in unplated segments). The horizontal myoseptum was not used to define the midline because both the Ma and Mp lines are dorsal to the horizontal myoseptum in the body segments around the pectoral fin. Although neuromasts are likely located in the epithelium over the plates, we state that neuromasts are located ‘on’ plates for simplicity.

All microscopy was performed using a Nikon Eclipse 80i microscope (Nikon Instruments Inc., Melville, NY, USA) with FITC/Texas Red filter cubes and manual stage and focus. Images were recorded using a Photometrics Cool-snap ES2 camera (Photometrics, Tucson, AZ, USA), then pseudocolored and uniformly enhanced for brightness and contrast with NIS-Elements imaging software (Nikon Instruments Inc., Melville, NY, USA). Composite (whole body and whole flank) pictures were stitched together using Adobe Photoshop.

### *Eda* gene expression

We made cDNA from developing PB and JP fry by collecting clutches of fish at 4 weeks post-hatching (after plates have begun to calcify). RNA was isolated separately from whole trunk (all tissue posterior to the operculum) of 12 individuals from each population using TRIzol (Invitrogen, Carlsbad, CA, USA), then was reverse transcribed into cDNA using the SuperScript III kit (Invitrogen, Carlsbad, CA, USA). Relative expression of *Eda* in JP and PB fish was determined by performing qPCR on these cDNA samples (Forward: GGAGAGGGTCATGAGGAGAAGTT; Reverse: GTTATCCTGTGTGGCATGCAA), with 3 technical replicates each of 12 biological replicates for each gene in each population. qPCR reactions were carried out and analyzed as described previously [50] using *Eef1b2* as the reference gene (Forward: CCGCTGGTACAACCACATCA; Reverse: ACTGACCCAGAGGCTTCTTCAC), and calculating primer efficiencies based on a standard curve made from serial dilutions of a pool of JP and PB cDNAs. Values reported are *Eda* expression as a percentage of reference gene expression.

### *Eda* transgenics

To make transgenic fish, we constructed a plasmid in which the expression of the JP allele of the *Eda* cDNA was driven by the human cytomegalovirus (CMV) promoter, with Tol2 sites [51] for increased efficiency of transgenesis. We used the CMV promoter because Colosimo *et al*. [39] previously demonstrated that expression of the mouse *Eda* gene under the control of the same promoter leads to an increase in number of plates in low plated fish. Constructs were generated by first amplifying the complete *Eda* coding region from JP trunk cDNA (F primer: ATGACACGCGACGGTTCA; R primer: TCAGTTTTGTCCAGCAGATGGA) and cloning it into the pCR-2.1 vector using a TOPO-TA cloning kit (Invitrogen, Carlsbad, CA, USA). This sequence has two variant nucleotide residues compared with the canonical ‘complete *Eda* allele’ shared by most marine sticklebacks that was previously described [39]. Specifically, there was a substitution of a T with a C at bp 95, leading to an amino acid substitution from V to A at position 32, and a substitution of a G with an A at bp 273, leading to a synonymous change. We targeted the A1 splice form, because the *Eda-A1* and *Eda-A2* splice forms bind different receptors in mammals [52], and Colosimo *et al*. [39] used mouse *Eda-A1* to demonstrate that *Eda* caused ectopic plate formation. To assemble the CMV:*JP*-*Eda-A1* construct in the Tol2 backbone, we amplified the CMV promoter from the p5E-CMV plasmid in the zebrafish Tol2Kit [53] using a forward primer that contained a 15 bp match with the Tol2 backbone (F primer: ATCACCGGGGGATCCAGGCCTCTTCGCTATTACG; R primer: TCTATAGTGTCACCTAAATCAAGC), amplified *Eda* from the plasmid containing the JP *Eda-A1* cDNA using primers that contained 15 bp matches with the 3′ end of the CMV promoter (F primer: AGGTGACACTATAGACCACCATGACACGCGACGGTTCA) and the 5′ end of the polyA tail in the Tol2 backbone (R primer: TGGATCATCATCGATTCAGTTTTGTCCAGCAGATGGA), digested the T2-hsp:EGFP plasmid (gift from Tim Howes and David Kingsley, Stanford University, Stanford, CA, USA) with BamHI and ClaI to remove hsp:EGFP, and combined all three components with InFusion (Clontech, Mountain View, CA, USA). We sequenced the final plasmid to verify all components had expected sequences. Single-cell stickleback embryos were each injected with approximately 1 nl of a solution containing 250 ng of the CMV:JP-*Eda* plasmid, 350 ng RNA encoding the Tol2 transposase enzyme (transcribed *in vitro* using the mMessage mMachine SP6 kit; Ambion, Austin, TX, USA), and 0.1% phenol red (Sigma-Aldrich, St. Louis, MO, USA). Microinjection was carried out as previously described [54].

We generated two populations of low plated *Eda* transgenics: PB pure crosses as well as PB x JP F3 hybrids. Parents for PB crosses were from wild-caught and/or lab-reared stocks. Parents for the PB x JP low plated F3 fish were F2 offspring from the *in vitro* cross of a PB female to a JP male. Low plated PB x JP F2s carrying the PB chromosome in the 7 Mb around the *Eda* locus were identified by genotyping with microsatellite markers in *Eda* (*Stn382* at 12.8 Mb [39]) and in flanking regions (*Stn47* at 16.33 Mb [55] and a newly designed marker at 9.01 Mb (Forward primer: GCCATTAGCCAAGGACTATGC; Reverse primer: CCTCTCTGTCCTTCTGTCATCC)). F2s that were found to have only PB (low plated) alleles in this region were crossed to generate low plated F3s.

When injected fish were at least 25 mm in standard length, they and their uninjected siblings were stained with calcein. Any injected fish that had more plates on either side than the highest number of plates on any of its uninjected siblings was identified as a putative transgenic. We looked independently at the number of plates on each side, rather than total number of plates, because we observed a difference in plate number on opposing sides of individual transgenic fish, likely owing to the mosaic integration of the transgene. A total of 11 independent PB clutches yielding 135 surviving fish were screened to identify 27 transgenics. A total of 12 independent PB x JP F3 clutches yielding 40 surviving fish were screened to identify 13 transgenics.

### Statistical analysis

All statistics were performed in R ( http://www.R-project.org). We used Kruskal-Wallis tests to evaluate *Eda* qPCR data as well as the effect of transgene and genetic background on plate number. The *P* values reported in the text have been corrected for multiple comparisons using Bonferroni correction. Data are reported as means ± standard error. Fisher’s exact test was used to analyze the relationship between plates and neuromast distribution during development and as a result of transgenic manipulation. We used the Test of Equal or Given Proportions to assess differences in the percentage of segments with dorso-ventral neuromasts in control versus transgenic fish and between genetic backgrounds.

## Results and discussion

### Developmental correlation between lateral plate formation and neuromast distribution

To examine whether there was a developmental correlation between the formation of the lateral plates and the distribution of neuromasts, we used repeated calcein staining to follow plate formation across the development of individual fish from a completely plated marine population (JP). Plate formation in this population follows the pattern described previously [56]. The first plates calcify near the Ma/Mp boundary (Figure 2A); additional plates are then added rostrally and caudally (Figure 2B). A second set of plates begins to calcify near the caudal fin (Figure 2C), and additional plates are then added to each set of plates until they join in the middle into a continuous series of plates (Figure 2D).

To determine whether the distribution of neuromasts along the flank is associated with the development of the lateral plates, we used double staining with calcein and DASPEI to track the temporal correlation between plate formation and neuromast appearance in two completely plated marine populations (JP and MC). At hatching (roughly 6 to 7 mm standard length), fry from both populations have roughly one neuromast per body segment down the length of the trunk (Figure 2E). Developing plates begin to calcify directly around the single neuromast in that segment (Figure 2F). As the plates grow in height, additional neuromasts appear above and below the original neuromast (Figure 2G). As the plates near the caudal fin begin to calcify, the same process is repeated in those segments (Figure 2H). Eventually, the two nascent neuromasts in each segment remain, while the original neuromast is no longer visible (Figure 2I). While each fish appears to follow this general schedule of plate development and neuromast elaboration, the size of the fish at the beginning of plate formation and the exact order of plate addition do not appear to be highly stereotyped (Figure 2J). Within each fish, however, the correlation between plate addition and neuromast distribution through development is striking, as can be seen in a segment-by-segment overlay of plate presence and neuromast distribution across MC fish of a variety of sizes (Figure 2J). Specifically, in developing MC fish, the presence of a plate in Mp is strongly associated with a dorso-ventral distribution of neuromasts within that segment. The vast majority (99%) of unplated segments in Mp possess neuromasts located along the midline, whereas neuromasts in plated Mp segments are significantly more likely to be located off of the midline (80% of plated segments; Table 1; Fisher’s exact test, *P* <0.0001). Given the tight spatial correlation between the appearance of a neuromast and the beginning of plate calcification, it is tempting to speculate that neuromasts provide a location cue for plate formation, as has been hypothesized for dermal bones in the heads of other fishes (see [43,57] for reviews). Testing this hypothesis will require the identification of plate precursor cells, as well as development of a method for ablating neuromasts that leaves those plate precursor cells intact.

**Table 1:** Plate presence and neuromast distribution are correlated through development

	**Dorso-ventral**	**Midline**
Plate	289^a^	72^a^
No plate	3^a^	272^a^

^a^Combined segment totals (average 25.5 segments per fish) for MC fry at a range of stages in plate development (n = 31 fish).

### Transgenic expression of *Eda* reveals pleiotropic effects on lateral plate formation and neuromast distribution

Our developmental analysis revealed a close relationship between plate formation and neuromast distribution. Previous work in our laboratory has shown strong phenotypic correlations between lateral plate presence and lateral line distribution observed across complete- and low plated populations [46] as well as in genetic crosses [38]. Together, these findings led us to hypothesize that *Eda* has a pleiotropic effect on both phenotypes. To test this hypothesis, we used transgenesis to manipulate *Eda* expression to drive ectopic plate formation in low plated sticklebacks. The completely plated (JP) *Eda* allele is dominant to the low plated (PB) *Eda* allele for plate formation [31,39], and we observed that overall *Eda* expression is 1.7 fold higher in the bodies of JP fish than in PB fish once plate calcification begins (relative expression JP: 0.96 ± 0.09 (n = 12); PB: 0.57 ± 0.05 (n = 12); Kruskal-Wallis χ^2^(1) = 9.3; *P* <0.0025). Thus, we used the broadly expressed human cytomegalovirus (CMV) promoter to upregulate *Eda* expression in PB fish. Injection of the CMV:*Eda* transgene into PB embryos caused formation of ectopic plates in both Ma and Mp (Figure 3B); we focus on Mp because the distribution of neuromasts in Mp (but not Ma) is linked to *Eda*[38]. Transgenic fish had significantly more plates in Mp than their uninjected siblings (uninjected controls: 0.05 ± 0.05 (n = 21); transgenic fish: 3.1 ± 0.6 (n = 19); Kruskal-Wallis χ^2^(1) = 19; *P* <0.0001). Transgenic fish also had significantly more segments in Mp that had dorso-ventral neuromasts (percentage of total segments with dorso-ventral neuromasts in uninjected controls: 0.3% (1/343 segments; n = 13 fish); transgenic fish: 7.7% (21/272 segments; n = 11 fish); Kruskal-Wallis χ^2^(1) = 22, *P* <0.0001). This shift in neuromast distribution was strongly associated with the presence of ectopic plates (Figure 3A,B; Table 2; Fisher’s exact test, *P* <0.0001). In transgenic fish, neuromasts were located dorsal and/or ventral to the midline in 44.4% of the segments with ectopic Mp plates, but in only 0.4% of unplated Mp segments. This result reveals that *Eda* has pleiotropic effects on both neuromast distribution and plate development.

**Table 2:** **Transgenic sticklebacks demonstrate that *Eda *** **expression affects both plate presence and neuromast distribution**

		**Transgenic**		**Control**	
**Population**		**Dorso-ventral**	**Midline**	**Dorso-ventral**	**Midline**
PB	Plate	20^a^	25^a^	1^b^	0^b^
	No plate	1^a^	226^a^	0^b^	342^b^
PB x JP F3	Plate	114^c^	6^c^	5^d^	0^d^
	No plate	0^c^	105^c^	0^d^	329^d^

Combined segment totals (average 25.5 segments per fish) for: ^a^transgenic fish in PB background (n = 11 fish); ^b^uninjected control siblings in PB background (n = 13 fish); ^c^transgenic fish in PB x JP F3 background (n = 12 fish); and ^d^uninjected control siblings in PB x JP F3 background (n = 15 fish). JP, Japanese Pacific Ocean marine; PB, Paxton benthic; PB x JP F3, Paxton benthic x Japanese Pacific Ocean marine F3 hybrid.

Although our results demonstrate that transgenic expression of *Eda* in the PB background does lead both to ectopic plate formation and to changes in neuromast distribution, there are relatively few ectopic plates in the Mp line of PB transgenic fish and only half of these plates have a dorsal-ventral distribution of neuromasts. These findings are consistent with previous results demonstrating that interactions between *Eda* and modifier loci in the PB background influence both plate number [31] and neuromast distribution [38]. To determine whether these modifiers were also affecting the *Eda* transgene, we generated CMV:*Eda* transgenics in a second low plated background: low plated PB x JP F3 hybrids. These hybrids were verified to be homozygous for PB alleles in at least a 7 Mbp region around *Eda,* but should have an assortment of PB and JP alleles at other plate modifier loci. In this F3 hybrid background, the average number of plates in Mp increased dramatically, from 0.4 ± 0.1 in uninjected controls (n = 26) to 12 ± 3 in transgenic fish (n = 12) (Kruskal-Wallis χ^2^(1) = 18, *P* <0.001). This was a significantly higher number of ectopic Mp plates than that observed in PB transgenics (Table 2; Kruskal-Wallis χ^2^(1) = 6.2, *P* <0.05). In F3 hybrids, the *Eda* transgene also significantly increased the number of Mp segments with a dorso-ventral distribution of neuromasts (percentage of total segments with dorso-ventral neuromasts in uninjected controls: 1.5% (n = 15); transgenic fish: 50.7% (n = 12); Kruskal-Wallis χ^2^(1) = 191, *P* <0.0001). Similar to the effect of the *Eda* transgene in the PB background, the significant shift in the distribution of neuromasts was strongly linked to being located on plates (Figure 3C-E; Table 2; Fisher’s exact test, *P* <0.0001). However, compared with the pure PB background, a significantly higher proportion of these ectopic plates had a dorso-ventral distribution of neuromasts in F3 hybrids: neuromasts were located dorsal and/or ventral to the midline on 95% of ectopic Mp plates in F3 transgenics versus 44.4% in PB transgenics (Table 2; Kruskal-Wallis χ^2^(1) = 51, *P* <0.0001). These results support previous genetic mapping studies demonstrating that modifier loci contribute to differences in these phenotypes between the JP and PB populations [31,38]. Additional genetic mapping and cloning experiments are required to determine whether the modifier loci act independently or have pleiotropic effects on the two phenotypes.

## Conclusions

These independent transgenic experiments provide strong support for the conclusion that *Eda* has pleiotropic effects on both the development of the lateral plates and the patterning of the lateral line. There are two possible mechanisms to explain the pleiotropic effects of *Eda* on these two phenotypes. *Eda* could have a direct effect on neuromast distribution that is independent of its effect on plate development. Alternatively, *Eda* could have an indirect effect on neuromast distribution, which is mediated by its influence on plate development. At this point, we cannot distinguish between these alternatives. However, studies in medaka and zebrafish demonstrate that the presence of dermal bone affects the patterning of some neuromasts [58], suggesting that similar mechanisms might occur in sticklebacks. Testing these alternative mechanisms would require ablation of the lateral plate precursor cells without disruption of the neuromasts in a transgenic background.

While the molecular genetic basis of lateral plate variation is now well-described [31,32,39], the selective forces that generate this variation remains a subject of debate [47,59]. Our results demonstrate that the repeated loss of lateral plates in freshwater stickleback populations is accompanied by a change in the lateral line sensory system. Thus, our work suggests that repeated selection for the low plated *Eda* allele in freshwater could also be the result of selection on the lateral line, rather than on lateral plates. Furthermore, many other phenotypes have been linked to the region around *Eda*, such as pelvic spine length [37], body shape [27,34,36], color [34], growth rate [28,29], salinity preference [30], and schooling behavior [60], all of which could also be targets of selection. Although associations between *Eda* genotype and survival and fitness in freshwater have been previously demonstrated [28,29,33,35], these experiments could not distinguish between the effects of *Eda* and linked loci. The transgenic fish generated for this study, and any stable transgenic lines obtained from them, will enable a rigorous analysis of the pleiotropic effects of *Eda* on both phenotypes and fitness. Determining which traits are caused by *Eda* itself or by other loci in tight linkage with *Eda* will enable future studies to determine both the agents and phenotypic targets of selection at this locus, thereby making key connections between genotype, phenotype and fitness [61,62].

## Abbreviations

CMV: human cytomegalovirus; DASPEI: 2-(4-(dimethylamino)styryl)-N-ethylpyridinium iodide; *Eda*: *Ectodysplasin*; JP: Japanese Pacific Ocean marine; Ma: Main trunk line, anterior; MC: Manchester Clam Bay marine; Mp: main trunk line, posterior; PB: Paxton benthic; PB x JP F3: Paxton benthic x Japanese Pacific Ocean marine F3 hybrid; QTL: quantitative trait locus.

## Competing interests

The authors declare that they have no competing interests.

## Authors’ contributions

MGM, AKG, and CLP conceived and designed the experiments, analyzed the data, and wrote the manuscript. MGM and AKG performed the experiments. All authors read and approved the final manuscript.
